# Identification of Novel Biomarkers of Homologous Recombination Defect in DNA Repair to Predict Sensitivity of Prostate Cancer Cells to PARP-Inhibitors

**DOI:** 10.3390/ijms20123100

**Published:** 2019-06-25

**Authors:** Daniela Criscuolo, Francesco Morra, Riccardo Giannella, Aniello Cerrato, Angela Celetti

**Affiliations:** 1 Institute for the Experimental Endocrinology and Oncology, Research National Council, CNR, 80131 Naples, Italy; daniela.cris@live.it (D.C.); francesco.morra@unina.it (F.M.); aniellocerrato@gmail.com (A.C.); 2 Department of Molecular Medicine and Medical Biotechnology, University “Federico II” of Naples, 80131 Naples, Italy; 3 Urology Surgery Unit, Antonio Cardarelli Hospital, 80131 Naples, Italy; riccardogiannella@libero.it

**Keywords:** genome instability, DNA damage response, synthetic lethality, BRCAness, CCDC6, biomarkers

## Abstract

One of the most common malignancies in men is prostate cancer, for which androgen deprivation is the standard therapy. However, prostate cancer cells become insensitive to anti-androgen treatment and proceed to a castration-resistant state with limited therapeutic options. Therefore, besides the androgen deprivation approach, novel biomarkers are urgently required for specific targeting in this deadly disease. Recently, germline or somatic mutations in the homologous recombination (HR) DNA repair genes have been identified in at least 20–25% of metastatic castration-resistant prostate cancers (mCRPC). Defects in genes involved in HR DNA repair can sensitize cancer cells to poly(ADP-ribose) polymerase (PARP) inhibitors, a class of drugs already approved by the Food and Drug Administration (FDA) for breast and ovarian cancer carrying germline mutations in *BRCA1/2* genes. For advanced prostate cancer carrying Breast cancer1/2 *(BRCA1/2)* or ataxia telengiectasia mutated (*ATM*) mutations, preclinical studies and clinical trials support the use of PARP-inhibitors, which received breakthrough therapy designation by the FDA. Based on these assumptions, several trials including DNA damage response and repair (DDR) targeting have been launched and are ongoing for prostate cancer. Here, we review the state-of-the-art potential biomarkers that could be predictive of cancer cell synthetic lethality with PARP inhibitors. The identification of key molecules that are affected in prostate cancer could be assayed in future clinical studies to better stratify prostate cancer patients who might benefit from target therapy.

## 1. Mechanism of Action of PARP-Inhibitors and Rationale for Their Inclusion in Clinical Settings

The human genome is constantly exposed to endogenous and exogenous genotoxic stress. To preserve the genome integrity, eukaryotic cells have evolved a complex array of DNA repair pathways [1] including base excision repair (BER), nucleotide excision repair (NER), and mismatch repair (MMR) pathways that repair the damage limited to a single DNA strand as single strand breaks (SSBs) or base modification. The DNA double strand breaks (DSBs) can be repaired by homologous recombination (HR), an error free mechanism that makes use of the sister chromatid as a template, or by non-homologous end joining (NHEJ)—an error prone mechanism that does not use a template to connect the broken ends. Molecular defects in HR DNA repair, promote NHEJ as the mechanism of DSBs DNA repair. This leads to genomic instability and cancer, and increases the susceptibility of cells to pharmacological inhibition of DNA repair enzymes, a phenomenon called synthetic lethality [2]. The PARP-inhibitors represent a class of drugs designed to exploit synthetic lethality as therapeutic strategy for the treatment of cancers with HR DNA repair deficiency. Poly(ADP-ribose) polymerases (PARPs) are a family of enzymes that catalyze the NAD+-dependent ADP-ribosylation of the target protein [3]. Poly(ADP-ribose) polymerase (PARP)-1, the best-characterized member of the PARP family, plays a crucial role in the repair of DNA single strand breaks (SSBs). In particular, PARP-1 orchestrates the recruitment of repair proteins at DNA break-sites. PARP-inhibitors compete with NAD+ for binding to the catalytic domain of PARP, inhibiting the catalytic activity of PARP-1 and inducing the accumulation of unrepaired SSBs that degenerate into the more lethal DSBs [4,5]. PARP-inhibitors are also able to trap PARP1 at the DNA damage sites, preventing DNA replication and transcription with cytotoxic effects [6]. Cells that harbor defects in HR repair genes treated with PARP-inhibitors can repair the resulting DSBs only through NHEJ, leading to genome instability and cell death. The efficacy of PARP-inhibitors has been well established for breast and ovarian cancers with germline *BRCA1/2* mutations. Recently, several studies have proposed the use of PARP-inhibitors in additional types of tumors including prostate cancer [7].

## 2. Rationale for Use of Poly(ADP-Ribose) Polymerase Inhibitors in Treatment of Prostate Cancer

Prostate cancer (PCa) is the fourth most common tumor type worldwide and is the most frequent cancer among men in Europe [8]. Most prostate cancer patients have localized disease at the time of diagnosis. Patients with localized PCa are managed with surgery, radiation therapy, and/or active surveillance. However, approximately 10% of men with localized disease at the time of diagnosis relapse with a metastatic disease [9]. The standard treatment for metastatic PCa is androgen deprivation therapy (ADT), which interferes with androgen signaling. Androgens are the male sex steroid hormones that, upon binding to the androgen receptor (AR), promote the initiation, growth, and progression of prostate cells. However, in older men suffering from PCa, androgens play an oncogenic function that occurs upon reprogramming of the transcriptome [10,11]. Despite the efficacy of androgen-deprivation therapy, most hormone-sensitive patients develop a stage of castration-resistant prostate cancer (CRPC) following ADT [12]. AR antagonists and the CYP17A inhibitor abiraterone are used successfully in CRPC patients, providing stabilization for about 18 months [13]. Taxanes are also approved for mCRPC [14]. Although considerable improvements in progression-free survival (PFS) and overall survival (OS) have been achieved, none of the therapeutic approaches explored for CRPC appeared to be effective. Hence, there is an urgent need to identify alternative systemic approaches.

In the CRPC setting, the response rate to conventional chemotherapy was highly variable in different studies, possibly due to patient heterogeneity, but also to the plasticity of cancer cells and the different types of somatic alterations occurring in the AR geneconsisting of AR genomic amplification, mutation, and duplication of an enhancer upstream of AR- that increases its expression. This can be also caused by AR antagonist treatment [15,16].

An additional castration-resistant mechanism is related to the expression of the AR splice variants, such as the AR-V7. This variant lacks the ligand binding domain and acts as a repressor of suppressive genes to support the castration resistant PCa growth [17,18].

Prostate cancer patients with the same histology and similar clinical measurements have different molecular profiles. However, CRPC is generally characterized by genomic instability, and mutations in DNA repair genes are enriched in the lethal metastatic disease [12,19]. In particular, the alterations in the *BRCA2* gene are correlated with a bad response to systemic therapy and a poor prognosis [20]. Defective DNA repair enhances tumor heterogeneity and promotes tumor progression.

Genome instability depending on DDR defects might favor the selection of resistant clones in ADT patients, leading to a castration resistant state. However, a defect in DNA repair molecules can also lead to a better management of these aggressive tumors on the basis of the synthetic lethal effect exerted by drugs like the PARP-inhibitors [21,22]. Thus, the genomic alterations in the homologous recombination DNA repair pathways can guide patient stratification and be used to tailor personalized treatments.

## 3. DNA Repair Deficiency and PARP-Inhibitors Response in Prostate Cancer

**Figure 1:**
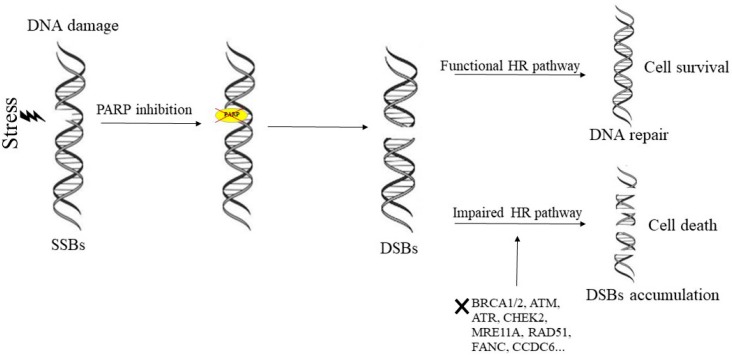
Synthetic lethality of PARP-inhibitors in HR-deficient tumors. Several stress can generate the single strand breaks (SSBs) that are repaired by poly(ADP-ribose) polymerases (PARPs) through the BER pathway. PARP inhibition prevent the repair of SSBs, resulting in the generation of double strand breaks (DSBs). The DSBs are repaired in cells through the functional HR-mediated DNA repair pathway, but in the presence of impaired HR pathway the DSBs cannot be effectively repaired resulting in DSB accumulation, genomic instability, and cell death.

Prostate cancer patients carrying germline mutations in HR DNA repair genes have been reported to have a higher Gleason score, advanced stages, and globally a worse prognosis with lower OS compared with non-carrier patients [23]. However, whereas only a minority of prostate cancer patients harbor germline mutations, about 11.8% in metastatic prostate cancer and about 4.6% in localized prostate cancer, many sporadic CRPCs carry genetic- and epigenetic-mediated defects in the homologous recombination pathway (Figure 1). Several somatic mutations have been identified in *BRCA1*, *BRCA2*, *FANC*, *ATM*, *CHEK2*, *MRE11A*, and *RAD51* genes in CRPC in about 23% of cases [24,25]. In a recent genome analysis, by comparing sequencing data obtained from castration-sensitive and castration-resistant prostate cancer, *BRCA2* was the most frequently mutated, occurring in 12.7% of cases [26]. The analysis of other DNA repair genes showed aberrations in 22.7% of patients, with *ATM* and *BRCA1* having the most frequent alterations in 19.3% of patients. Mutations in *CDK12*, *FANCA*, *RAD51B*, and *RAD51C* were also recorded in 3.4% of patients [27]. A list of altered BRCA-like genes that predict PARP inhibitor sensitivity was recently reported [28]. Here, we summarize the BRCA-like genes that have been found to be related to prostate cancer, predicting sensitivity to PARP inhibitors (Table 1). Several types of cancer genomic sequencing, such as germline sequencing, somatic sequencing, cell-free DNA assays, and circulating tumor cell assays of localized and advanced prostate cancers, have been reported [29,30,31].

Then, prostate cancer patients with HR defects are at high risk of an aggressive disease. Patients who are carriers of *BRCA2* germline mutations showed an increased risk ranging from 5.0- to 8.6-fold, and an absolute risk of 15% of developing prostatic adenocarcinoma [25,32]. In a further analysis, high rates of prostate cancer progression from localized to systemic disease were observed in a cohort of patients carrying germline mutations in the *BRCA1/BRCA2* genes (*n* = 79). The patients with *BRCA1/2* germline mutations have a 23% local failure rate in contrast to the 7% local failure rate among the non-carriers [33]. Additional studies have validated the association between germline defects in *BRCA1/2* genes and increased aggressiveness.

Overall, these emerging data suggest a possibility of a molecular stratification and of the use of PARP-inhibitors in mCRPC patients when DNA-repair defects are detected. In a multicenter Phase II clinical trial (TOPARP), the association between somatic DNA repair gene mutations and the response to PARP-inhibitor Olaparib has been investigated [34]. Fifty patients with mCRPC that progressed after one or two cycles of chemotherapy were enrolled to receive Olaparib at a dose of 400 mg twice per day. Patients with previous exposure to platinum were excluded. Whole-exome sequencing and transcriptome analysis were performed on DNA from freshly frozen tumor biopsy tissue obtained before treatment, whereas the germline whole-exome sequencing was performed on DNA from saliva samples. Carriers of defects in genes involved in DNA repair mechanisms were identified in two groups of patients. In this study, the overall response rate was 33% and the median overall survival was 10.1 months. Of the biomarker-positive patients, 88% had a good response to Olaparib. These patients also presented a prolonged PFS and OS when compared to the biomarker negative population (9.8 *vs*. 2.7 months and 13.8 *vs*. 7.5 months, respectively). *BRCA2* was the most frequent altered DNA repair gene, and the patients carrying these mutations were responsive to Olaparib. Four out of the five patients with *ATM* abnormalities and the patients harboring less common DNA repair genes mutations, such as in *PALB2*, *FANCA*, and *HDAC2*, also responded to Olaparib. One patient harboring *MLH3* loss and one carrying the *ATM* mutation did not respond to Olaparib. Conversely, only two (6%) of the biomarker-negative patients responded to PARP-inhibitors treatment. No correlation was detected between a *PTEN/ERG* mutational status and Olaparib response. Overall, this trial demonstrated the utility of PARP inhibition as a therapeutic strategy in mCRPC patients with somatic mutations in HR DNA repair genes, confirming the synthetic lethal effect exerted by PARP-inhibitors in carriers of DNA HR defects also in sporadic tumors [35]. In the sentinel TOPARP-A study, evaluating the effects of PARP-inhibitor Olaparib in unselected mCRPC, 14 of 16 patients with DDR defects demonstrated a response (compared to 2 of 33 patients with an intact DDR), all with *BRCA2* defects [21,34].

It is possible that the mCRPC patients that are responsive to PARP-inhibitors treatment with an intact DDR might carry mutations in and/or express altered level of proteins, involved in HR DNA repair and not yet identified.

Cellular models depleted of CCDC6 behave as BRCA-like cells, with a defect in HR DNA repair, resistance to standard chemotherapy, and sensitivity to PARP-inhibitors [36,37]. Altered levels of *CCDC6* gene product in tumoral cells have been ascribed to altered turnover regulated by the FBXW7 ubiquitin ligase and by the deubiquitinase USP7 [38,39,40]. CCDC6 attenuation in prostate cancer cells confers sensitivity to Olaparib, independent of their castration resistance status [41]. Then, CCDC6 and USP7 may be predictive biomarkers for the combined treatment of USP7 and PARP-inhibitors in advanced prostate cancer.

However, until phase III trials are completed, the genes other than *BRCA1/BRCA2* that could be considered predictive for PARP inhibitor response in advanced prostate cancer will likely remain unknown [41,42]. At the moment, several trials are underway to test the efficacy of different PARP inhibitors at different stages of prostate cancer with known/suspected deleterious mutations in DNA repair genes.

## 4. Ongoing Prostate Cancer Clinical Trials Involving PARP Inhibitors

Recent studies have evaluated several PARP inhibitors, including Rucaparib, Olaparib, Niraparib, Veliparib, and Talazoparib, for their PARP-trapping potency. Although PARP inhibitors have a similar ability to inhibit PARP catalytic activity, they display different trapping capacities [43,44]. Of the PARP inhibitors evaluated, Veliparib has the lowest trapping activity whereas Talazoparib is about a 100-fold more potent PARP trapper than Rucaparib, Niraparib, and Olaparib [43,44,45]. The different trapping potencies of PARP inhibitors appear to drive the PARP inhibitor cytotoxicity in the monotherapy setting, whereas this characteristic seems to be less relevant when the PARPi are used in combination with DNA-damaging agents [44]. The potency of PARP-trapping may be an important factor to consider when identifying the most appropriate PARP inhibitor and therapeutic regimen (single agent or combination) for cancer treatment. Distinct PARPi have different pharmacokinetic and pharmacodynamic properties that need to be considered for their use as a single agent or in combination. Niraparib shows a tumor exposure 3.3 times greater than plasma exposure in BRCA wildtype (wt) patient-derived ovarian cancer xenograft models compared to Olaparib. Pharmacodynamic analysis indicated that Niraparib is able to deliver ~90% of the PARP inhibition for 24 hours at steady state [46]. These findings indicate that the potent antitumor effects of Niraparib, particularly in BRCA wt tumor, could, at least partially, be attributed to their different pharmacokinetic properties.

The first clinical study involving PARP inhibitors in prostate cancer treatment was conducted at the Royal Marsden National Health Service (NHS) Foundation Trust (United Kingdom) and the Netherlands Cancer Institute (The Netherlands) in 2009 [47]. In this phase I trial, 60 patients with castration-resistant prostate cancer, carrying *BRCA1/2* mutations and refractory to standard therapies, were treated with escalating doses of Olaparib. This trial was followed by the multicenter Phase II clinical trial TOPARP in 2015, and the results were extensively discussed in the previous paragraph [34]. Besides Olaparib, several PARP inhibitors, such as Rucaparib, Niraparib, and Talazoparib have been included in ongoing clinical trials for the treatment of prostate cancer. All the mentioned PARP inhibitors have received FDA approval in breast and ovarian cancer: Olaparib (Lynparza, Astra Zeneca, Cambridge, UK) was first approved by the FDA as a third-line treatment for ovarian cancer carrying germline mutations in *BRCA* genes (*gBRCA*) in 2014, and for HER2-positive metastatic breast cancer in 2018; the PARP inhibitor Rucaparib (Rubraca, Clovis Oncology, Boulder, Colorado, Stati Uniti) was FDA approved as a third-line treatment for gBRCA-mutated ovarian cancer in 2016; the drug Niraparib (Zejula, TESARO Bio Italy S.r.l.) was first approved by the FDA as maintenance therapy in platinum-sensitive ovarian cancer in 2017; and the PARP inhibitor Talazoparib (Talzenna, Pfizer Italia S.r.l., ROMA, ITALY) was approved by the FDA for locally advanced or metastatic HER2-negative breast cancer with gBRCA mutations in 2018.

In prostate cancer, several studies examined different PARP inhibitors included alone, before or after prostatectomy, and/or in combination with the anti-androgen abiraterone and/or the corticosteroid prednisone. Olaparib has been included in two single-arm studies: BrUOG 337 (NCT03432897), for locally advanced prostate cancer (LAPC) prior to prostatectomy, and NCT03047135 for recurrent prostate cancer (rPCa) following prostatectomy, and then in the clinical trial NCT03012321 in combination with abiraterone, for metastatic prostate cancer that is castration resistant.

The PARP inhibitor Rucaparib has been included in three single arm studies: TRIUMPH (NCT03413995) for hormone sensitive metastatic prostate cancer, TRITON2 (NCT02952534) for castration resistant metastatic prostate cancer, and ROAR (NCT03533946) for non-metastatic castration resistant prostate cancer. Rucaparib is also being tested in the clinical trial TRITON3 (NCT02975934) that is evaluating Rucaparib versus abiraterone, enzalutamide, or docetaxel for mCRPC. Niraparib and Talazoparib have now been included in two single arm studies, Galahad (NCT02854436) and NCT03148795, respectively, both for metastatic prostate cancer resistant to castration [48,49,50,51].

The mentioned major ongoing clinical trials on prostate cancer involving PARP inhibitors are listed in Table 1. The evaluation of the mutational status of the genes involved in the DNA damage response (DDR) pathway, mainly *BRCA1/BRCA2* but also other DDR-genes, such as *ATM*, *CHEK1/2, FANCA*, and *RAD51*, is being considered in all the listed studies (Table 2).

The FDA granted orphan drug designation to Veliparib for the treatment of advanced squamous NSCLC according to the drug’s manufacturer in 2016. In 2017, Abbvie reported that Veliparib failed to improve outcomes in triple negative breast cancer and NSCLC trials. Regardless, a Phase 2 clinical trial (NCT01576172) including abiraterone acetate and prednisone with or without Veliparib is ongoing in patients with mCRPC.

Notably, the translational potential of PARP-1 PET imaging agent to test PARP1 expression has been examined in a preclinical model of ovarian cancer. Future clinical trials to analyze PARP1 expression might be considered in prostate cancer as a method to stratify patients for PARP inhibitor therapy and to limit resistance caused by low enzyme expression [52].

## 5. Sensitivity to PARP-Inhibitors Induced in Prostate Cancer with Apparent Integrity of Homologous Recombination Machinery

Prostate cancer is a heterogeneous disease and the identification of predictive biomarkers for patient stratification and personalized treatment is an unmet need. 

The use of PARP-inhibitor drugs will dramatically change the management of CRPC and clinicians need to urgently add novel tests to routine biopsy to identify patients suitable for PARP-inhibitors treatment. The ideal biomarker to determine sensitivity to PARP inhibitors would be recombination deficiency, but unfortunately no such biomarker exists and different strategies could be used.

Recently, a randomized placebo controlled Phase II trial compared abiraterone alone with abiraterone plus Olaparib for the treatment of 142 men with mCRPC, showing a trend favoring abiraterone plus Olaparib over abiraterone alone, with no associations between homologous recombination status and treatment group [53]. Since abiraterone plus Olaparib improved the radiographic PFS compared to abiraterone alone, these results suggest that the combination of androgen-receptor (AR) targeted therapy with PARP inhibitors targeted therapy may result in a new type of synthetic lethality [54]. Then, the inhibition of the AR signaling pathway with abiraterone may induce a DNA repair deficiency status (a so-called BRCAness state), a condition that could be investigated using concurrent PARP blockade with Olaparib [55,56,57,58,59,60]. These preclinical data also support the idea that the androgen receptor may promote DNA repair, particularly through activating the transcription of DNA-dependent protein kinase [61]. Larger prospective and biomarker stratified randomized trials are needed to support the hypothesis of this novel synthetic lethality involving the interplay between androgen receptor signaling and PARP functions [62].

Furthermore, P5091, the inhibitor of the de-ubiquitinase USP7, has been reported to be able to reduce protein levels of both full-length AR and AR-V7 spliced isoform, whose expression is related to the appearance of castration resistance. This effect might be ascribed to USP7 deubiquitinase stabilizing the AR-V7/AR heterodimers, impairing the AR-dependent transcription in cancer cells [39].

However, the deubiquitinase USP7 has many substrates [63] including several tumor suppressors and CCDC6, the tumor suppressor [64,65] whose reduced levels impair HR DNA repair and sensitize cancer cells to treatment with PARP inhibitors, as reported in several malignancies [36,37,38,39,40]. In prostate cancer, targetable levels of USP7 and CCDC6 have been detected in a wide series of prostate tumor biopsies via IHC staining [41]. Thus, CCDC6 and USP7 might represent novel predictive biomarkers for the combined treatment of the USP7 inhibitors and PARP inhibitors in both hormone-sensitive and androgen-resistant prostate tumors. Combined treatment with USP7 inhibitors and PARP inhibitors may be able to target the AR and DDR pathways, inducing a synthetic lethal effect [39,66]. However, the DUB inhibitor P5091, which has exhibited favorable preclinical activity in several tumors, has yet to be advanced to clinical trials [67,68].

Finally, as suggested by preclinical investigations, novel combinatorial strategies including immune checkpoint inhibitors, epigenetic and/or DDR targeting agents are envisaged as new therapeutic approaches in genito-urinary malignancies [41,69,70].

## 6. Combinations of PARP-Inhibitors with Immune-Checkpoint Inhibitors

Evidence indicates a synergistic therapeutic effect due to the combination of DDR inhibitors (DDRi) with immune-checkpoint inhibitors (ICIs) [71,72]. The rationale that supports the combination of these two inhibitors is related to the defect in DDR proteins contributing to genome instability with the accumulation of DNA damage and the enhancement of the tumor mutational burden (TMB) that ultimately leads to the generation of neoantigens, presented on the cancer cell surface in a complex with the major histocompatibility complex (MHC) class I [73]. The high tumor mutational burden and the induced immunogenicity of cancer cells should elicit an antitumor immune response mediated by the activation of T-cell lymphocytes that is ineffective in cancer patients [74]. Defects in DNA HR typically results in a higher number of mutational events in the cancer cell and the consequent generation of neoantigens that might explain the improved response to immunotherapy in HR defective tumors. A high burden of mutations in tumors is predictive for the enhanced expression of neoantigens and for better responses to ICIs in preclinical models [75]. Based on this evidence, tumors with defects in HR DNA repair, genomic instability and copy-number aberrations are responsive to both PARP inhibitors and ICIs [76]. Furthermore, the accumulation of DNA damage due to a defect in repairing the DSBs in the cancer cells may result in the induction of stimulator of interferon genes (STING), an innate immune signaling that is activated by cytosolic DNA as it occurs upon viral infection. STING mediates the release of type I interferons from cancer cells, leading to the activation of T cells for the innate immune recognition of immunogenic tumors [77].

The activation of STING in cancer cells is also associated with upregulation of the immunosuppressive molecule PD-L1, which, upon interaction with PD1, prevents the attack of cancer cells by the immune system [78]. Therefore, since immune therapy is not always effective, combinatorial strategies can be used to enhance its efficacy. Preclinical studies have revealed that PARP inhibition upregulates PD-L1 expression via inactivation of GSK3β and consequently attenuation of anti-tumor immunity [74]. PARP inhibition has been shown to enhance CHK1- and interferon-dependent expression of PD-L1 in cancer cell lines depleted of BRCA2 or Ku70/80 [79]. The effect exerted by the homologous recombination defects (HRD) and/or DDR inhibitors might create immunological vulnerabilities [80].

Thus, targeting HRD or even HR-proficient tumors with PARP inhibitors or other DDR inhibitors in combination with anti-PD-1/PD-L1 antibodies represents a new therapeutic strategy, also considering the non-overlapping toxicities of these drugs. Combinations of DDR and ICIs inhibitors are currently being tested in clinic for several tumors. Even though PCa has shown generally less neoantigens than other tumors, it was encouraging that durvalumab (ICI) was able to enhance the PFS of Olaparib (DDRi) in patients with mCRPC. T cell activation was shown to be associated with improved PFS, and early data from a phase II trial (NCT02484404) testing the combination of Olaparib and durvalumab in patients with mCRPC demonstrated an acceptable toxicity profile together with serum prostate-specific antigen responses in 12 of 17 patients (71%), with a median PFS of 16.1 months for all patients who received the combination [81]. Importantly, biochemical responses are related to the occurrence of mutations in DNA repair genes [81]. At this stage of the studies, it is difficult to define to what extent the anti-PD-1 or anti-PD-L1 antibodies are able to enhance the effects of PARP inhibition in patients with germline *BRCA1/2* mutations. Since few data support the use of checkpoint inhibitors in prostate cancer, further studies are required to determine the tumor types and the molecular profiles of cancer cells for which the combination of the two types of inhibitors will ultimately have biological and clinical efficacy (Table 3).

In prostate cancer, besides PARP inhibition, the targeting of DDR molecules such as ATM/ATR might be explored also in combination with chemotherapy, immunotherapy, and AR-targeted agents. The combination of an ATR inhibitor and an AR-antagonist has recently shown improved efficacy in a prostate cancer xenograft model. A novel ATR kinase inhibitor BAY 1895344 has been tested in association with external beam radiation therapy, PARP inhibitor or anti-androgen (AA) therapy (http://cancerres.aacrjournals.org/content/78/13_Supplement/321).

The combination of small molecule PARP and Nicotinamide phosphorybosyl transferase (NAMPT) inhibitors may represent a rational combinatorial strategy since the NAMPT blocks the rate limiting enzyme in the production of NAD+, a necessary substrate of PARP. Then, the cell killing phenotype of these combined drugs includes depletion of NMN and NAD+, diminished PAR activity, and increased the DNA damage and apoptosis. Combination of PARP inhibitors and NAMPT inhibitors in vivo may result in tumor regression, delayed disease progression, and increased survival. In the future, the use of these drugs in combination may represent a possible therapeutic option in the late treatment of prostate cancer [87].

## 7. Conclusions

International multi-omic studies have increased our knowledge about molecular drivers for cellular transformation and prostate tumor progression. In turn, these discoveries can offer novel opportunities for biomarker-driven patient management, for targeted therapy, and overall for patient prognosis. Lately, the most exciting discovery for prostate cancer has been the discovery of a high prevalence of DDR defects [88]. However, for prostate cancer patients, it is still unclear which DDR defects may induce sensitivity to PARP inhibitors and/or other agents seeking to create a synthetic lethal scenario. The DDR defects might promote genomic instability and facilitate the selection of resistant prostate cancer cells. Given the aggressive behavior of DDR-deficient prostate cancer, there is an urgent need to develop strategies to recognize this subset of affected patients early in their disease course to improve the disease outcome using personalized treatment [89]. Almost 30% of patients with CRPC carry germline or somatic alterations in DDR genes. Thus, the treatment with PARP-inhibitor drugs may represent a real therapeutic option for a large percentage of patients with CRPC harboring DNA repair gene mutations [89]. 

In summary, the analysis of recent studies promotes the use of PARP inhibitors as a new therapeutic strategy for CRPC tailored to the genomic characteristics of the tumor or the specific expression of proteins involved in HR DNA repair mechanisms. Besides the response to PARP inhibitors based on a native synthetic lethality, combinatorial approaches might enhance the vulnerability of cancer cells to PARP inhibitors by inducing a synthetic lethal effect. Emerging data about HR DNA repair mechanisms in CRPC suggest that in a context of HR integrity, ADT can affect HR prior to the development of castration resistant status, and that the combination of PARP inhibitors with ADT could be beneficial in advanced or high-risk prostate cancer [28,53]. The inhibition of USP7, able to affect the stability of the AR isoforms but also that of proteins like CCDC6 involved in HR impairment, might be able to sensitize hormone-sensitive and hormone-resistant prostate carcinoma to PARP inhibition [41].

The availability of a larger amount of biological data and the identification of novel biomarkers predictive of the response to PARP inhibitors will lead to the selection of the best therapeutic approach in a disease as heterogeneous as CRPC.

## Figures and Tables

**Table 1:** DNA repair genes that predict PARP-inhibitors sensitivity.

Gene	Functions in DNA Repair	Evidence for PARP Sensitivity in Prostate Cancer Patients	Reference
*BRCA1*	Phosphoprotein that assists in 5′ to 3′ resection of DSBs, loading of RAD51	NCT01682772	[34]
*BRCA2*	Phosphoprotein that assists with RAD51 loading on DNA	NCT01682772	[34]
*ATM*	Serine/threonine protein kinase involved in repair of DSBs	NCT01682772	[34]
*FANC A/F*	DNA repair protein involved in a post-replication repair	NCT01682772	[34]
*CHK2*	Serine/threonine protein kinase involved in repair of DSBs	NCT01682772	[34]
*RAD51B/C*	Assist the recruitment, stabilization, and loading of RAD51	NCT01682772	[34]
*CDK12*	Cyclin-dependent kinase that regulates the expression of genes involved in DNA repair	NCT01682772	[34]

**Table 2:** Clinical trials of PARP inhibitors in prostate cancer with defects in DNA repair genes.

PARP Inhibitor	ClinicalTrials.gov Identifier	Population	DNA Repair Genes	Treatment
Niraparib	NCT02854436 Galahad	mCRPC	*BRCA1/2, ATM, FANCA, PALB2, CHEK2, BRIP1,* or *HDAC2*	Niraparib (single-arm study)
Olaparib	NCT03432897 BrUOG 337	LAPC	*BRCA1, BRCA 2, ATM, CHEK1, CHEK2, FANCONIS ANEMIA (FANCL), HDAC2, PALB2, BARD1, BRIP1, CDK12, PPP2R2A, RAD51B, RAD51C, RAD51D, RAD54L*	Olaparib prior to prostatectomy (single-arm study)
	NCT03012321	mCRPC	*ATM, BRCA1, BRCA2, FANCA, PALB2, RAD51, ERCC3, MRE11, NBN, MLH3, CDK12, CHEK2, HDAC2, ATR, PMS2, GEN1, MSH2, MSH6, BRIP1,* or *FAM175A*	Abiraterone/Prednisone, Olaparib or Abiraterone/Prednisone + Olaparib
	NCT03047135	rPC	*ATM, BARD1, BRCA1, BRCA2, BRIP1, CDK12, CHEK1, CHEK2, FANCL, PALB2, PPP2R2A, RAD51B, RAD51C, RAD51D*	Olaparib following prostatectomy (single-arm study)
Rucaparib	NCT03413995 TRIUMPH	mHSPC	*BRCA1, BRCA2, ATM, CHEK2, NBN, RAD50, RAD51C, RAD51D, PALB2, MRE11, FANCA, FANCB, FANCC, FANCD2, FANCE, FANCF, FANCG, FANCI, FANCL, FANCM*	Rucaparib (single-arm study)
	NCT02952534 TRITON2	mCRPC	*BRCA1, BRCA2, ATM, BARD1, BRIP1, CDK12* *CHEK2, FANCA, NBN* *PALB2, RAD51, RAD51B* *RAD51C, RAD51D, RAD54L*	Rucaparib (single-arm study)
	NCT02975934 TRITON3	mCRPC	*BRCA1, BRCA2, ATM*	Rucaparib vs abiraterone, enzalutamide or docetaxel
	NCT03533946 ROAR	nmCRPC	*ATM, ATR, BARD1, BRCA1, BRCA2, BRIP1, CDK12, CHEK1, CHEK2, ERCC3, FAM175A, FANCA, FANCL, GEN1, HDAC2, MLH1, MRE11, NBN, PALB2, PPP2R2A, RAD51, RAD54L*	Rucaparib (single-arm study)
Talazoparib	NCT03148795	mCRPC	*BRCA1, BRCA2*	Talazoparib (single-arm study)

mCRPC: Metastatic Castration-Resistant Prostate Cancer, LAPC: Locally Advanced Prostate Cancer, rPC: Recurrent Prostate Cancer, mHSPC: Metastatic Hormone-Sensitive Prostate Cancer, nmCRPC: Non Metastatic Castration-Resistant Prostate Cancer.

**Table 3:** Clinical trials on the use of immune checkpoint inhibitors in prostate cancer.

ClinicalTrials.gov Identifier	Patients	Immune Checkpoint Inhibitors	Reference
NCT00323882	Metastatic hormone refractory prostate cancer	Ipilimumab	[82]
NCT00861614	Castration Resistant Prostate Cancer	Ipilimumab	[83]
NCT01057810	Metastatic Chemotherapy-Naïve Castration Resistant Prostate Cancer	Ipilimumab	[84]
NCT02054806	Advanced Adenocarcinoma	Pembrolizumab	[85]
NCT02312557	Metastatic Castration Resistant Prostate Cancer	Pembrolizumab	[86]

## Abbreviations

mCRPC	metastatic castration resistance prostate cancer
PARP	Poly (ADP-ribose) polymerase
DDR	DNA damage response and repair
FDA	food and drug administration
BRCA	Breast cancer
ATM	ataxia telengiectasia mutated
HR	homologous recombination
BER	base excision repair
NER	nucleotide excision repair
MMR	mismatch repair
NAD	nicotinamide adenine dinucleotide
SSBs	single strand breaks
DSBs	double strand breaks
NHEJ	non-homologous end joining
PCa	prostate cancer
ADT	androgen deprivation therapy
AR	androgen receptor
CRPC	castration resistant prostate cancer
PFS	progression free survival
OS	overall survival
FANCA	FA Complementation Group A
CHEK2	checkpoint kinase 2
MRE11	meiotic recombination 11 homolog 1
RAD51	recombinase 51
CDK12	cyclin dependent Kinase 12
PALB2	Partner and localizer of BRCA2
HDAC2	Histone deacetylase 2
MLH3	MutL Homolog 3
PTEN	Phosphatase and Tensin Homolog
ERG	ETS-Related Gene
CCDC6	coiled coil domain containing 6
FBXW7	F-box/WD repeat-containing protein 7
USP7	Ubiquitin-specific-processing protease 7 (USP7)
LAPC	Locally, Advanced Prostate Cancer
rPC	Recurrent Prostate Cancer
mHSPC	Metastatic Hormone-Densitive Prostate Cancer
nmCRPC	Non Metastatic Castration-Resistant Prostate Cancer
DDRi	DNA Damage Response inhibitors
TMB	tumor mutational burden
MHC	major histocompatibility complex
STING	stimulator of interferon genes
PD-1	Prorammed cell death protein 1
PD-L1	Ligand of PD-1
NSCLC	Non-Small Cell Lung Cancer
HNSCC	Head and Neck Squamous Cell Carcinoma
NAMPT	Nicotinamide phosphorybosyl transferase
NMN	Nicotinamide mononucleotide
